# Maternal fatty acid intake and human embryonic growth: the Rotterdam Periconception Cohort

**DOI:** 10.1007/s10654-024-01184-8

**Published:** 2024-12-11

**Authors:** Eleonora Rubini, Lenie van Rossem, Sam Schoenmakers, Sten P. Willemsen, Kevin D. Sinclair, Régine P. M. Steegers-Theunissen, Melek Rousian

**Affiliations:** 1 Department of Obstetrics and Gynecology, Erasmus MC, University Medical Center, Rotterdam, The Netherlands; 2 Department of Biostatistics, Erasmus MC, University Medical Center, Rotterdam, The Netherlands; 3 School of Biosciences, University of Nottingham, Sutton Bonington, Leicestershire, UK; 4 Division of Human Nutrition and Health, Wageningen University, Wageningen, The Netherlands

**Keywords:** Pregnancy, Fetal development, Omega 3, Polyunsaturated fatty acids, Supplements, Virtual reality

## Abstract

The required intake of macronutrients by women during the periconceptional period for optimal fetal growth is the subject of ongoing investigation. Intake of polyunsaturated fatty acids (PUFA) is positively associated with fetal neural development, growth velocity and birth weight. However, limited evidence indicates that PUFAs play a role in embryogenesis. We aim to investigate the associations between maternal PUFA dietary intake and first trimester embryonic volume (EV) and head volume (HV). In a prospective cohort study (2013–2020), 464 pregnant women at < 8 weeks of gestation were included. Maternal dietary intake of PUFAs, including omega 3 (docosahexaenoic acid, DHA and eicosapentaeonic acid, EPA) and 6, was obtained from food frequency questionnaires, and first trimester three-dimensional ultrasound examinations were performed to measure EV and HV using Virtual Reality techniques. More than 70% of the population had omega 3 intakes below recommendations. A higher intake of PUFAs was associated with a smaller embryonic HV/EV ratio after adjusting for confounders (EPA *p* = 0.012, DHA *p* = 0.015, omega 3 and 6 *p* < 0.001), but no associations were found with EV or HV alone. Omega 3 from fish oil supplements alone was not associated with embryonic growth. Strong adherence to a PUFA-rich dietary pattern was associated with a smaller embryonic HV/EV ratio (DHA and EPA-rich diet *p* = 0.054, PUFA-rich diet *p* = 0.002). It is important to increase awareness of the high prevalence of omega 3-deficiency among pregnant women, and the opportunity for prevention by increasing PUFA intake, thereby reducing the risks of adverse pregnancy outcomes which originate during the periconceptional period.

**Supplementary Information:**

The online version contains supplementary material available at 10.1007/s10654-024-01184-8.

## Introduction

The nutritional status of women during the periconceptional period has significant effects on maternal health, prenatal growth and, consequently, child physiological development and mental health [1–4]. Studies show that maternal dietary patterns with excessive or deficient intakes of macronutrients have profound effects on embryonic and fetal growth [5–14]. Impairments of growth at such early stages of development are risk factors for pregnancy complications and non-communicable diseases in adult life [15, 16]. We have previously demonstrated that growth delays and a smaller embryo during the first trimester are associated with a higher risk of miscarriages, lower mid-gestation estimated fetal weight and lower birth weight [17–19].

The nature of fatty acids (FA) in the mother’s diet is also relevant during pregnancy. Diets rich in saturated and trans FA are associated with pregnancy and fetal complications [20]. In contrast, polyunsaturated FA (PUFA) are known to confer general health benefits for offspring development, including brain maturation and body growth. Fetal synaptogenesis and neural development require membrane phospholipids which are synthesized from essential PUFAs, derived exclusively from the maternal diet. Indeed, docosahexaenoic acid (DHA) and eicosapentaenoic acid (EPA) are long chain omega-3 PUFAs which deposit in the fetal brain and retina [21]. Previous studies report that DHA and EPA are associated with a larger child brain volume [22], and lower levels of DHA and EPA are associated with postnatal neuropsychiatric diseases, cognitive impairments, age-related neurological diseases [23–25] and child metabolic issues [26, 27]. Yet, there is room for additional research as these observations are not always supported [28].

Studies also indicate that maternal intake of omega-3 PUFAs is essential for fetal growth and development. A higher intake of DHA and EPA is positively associated with fetal size, higher growth velocity of fetal weight, birth weight and duration of pregnancy [29, 30]. There is compelling evidence to indicate that the effect of omega-3 FAs is significant from late gestation and during lactation. However, there is also evidence that omega-3 PUFAs are important for first trimester trophoblast cell activity and embryonic stem cell cycle progression and neurogenesis, indicating they also play a role during early pregnancy; a time period that requires exploring [31, 32]. With the latest advances in ultrasound technology and imaging, there is the possibility to explore patterns of growth as early as the first trimester of pregnancy, which may predict any growth-related complications later during pregnancy [33–35].

Despite international health guidelines promoting the consumption of fish or fish oil supplements during the periconceptional period, their consumption remains poor in many populations and data about the side effects are largely unknown [36]. There is also ambiguous evidence of the advantage of fish oil supplements compared to FA from whole foods during pregnancy. We hypothesize that dietary intake of healthy FA during the periconception period positively influences early embryonic growth. This current study explores maternal dietary intake of FA (both quantity and quality) during the periconceptional period, with emphasis on omega 3 PUFAs, and investigates its associations with embryonic growth during the early stages of pregnancy using state-of-the-art ultrasound imaging technologies.

## Materials and methods

This study is embedded in the Rotterdam Periconception Cohort (Predict Study), a prospective tertiary hospital-based birth cohort study at the Department of Obstetrics and Gynecology of the Erasmus MC, University Medical Center, The Netherlands [37, 38].

### Study design

**Fig. 1:**
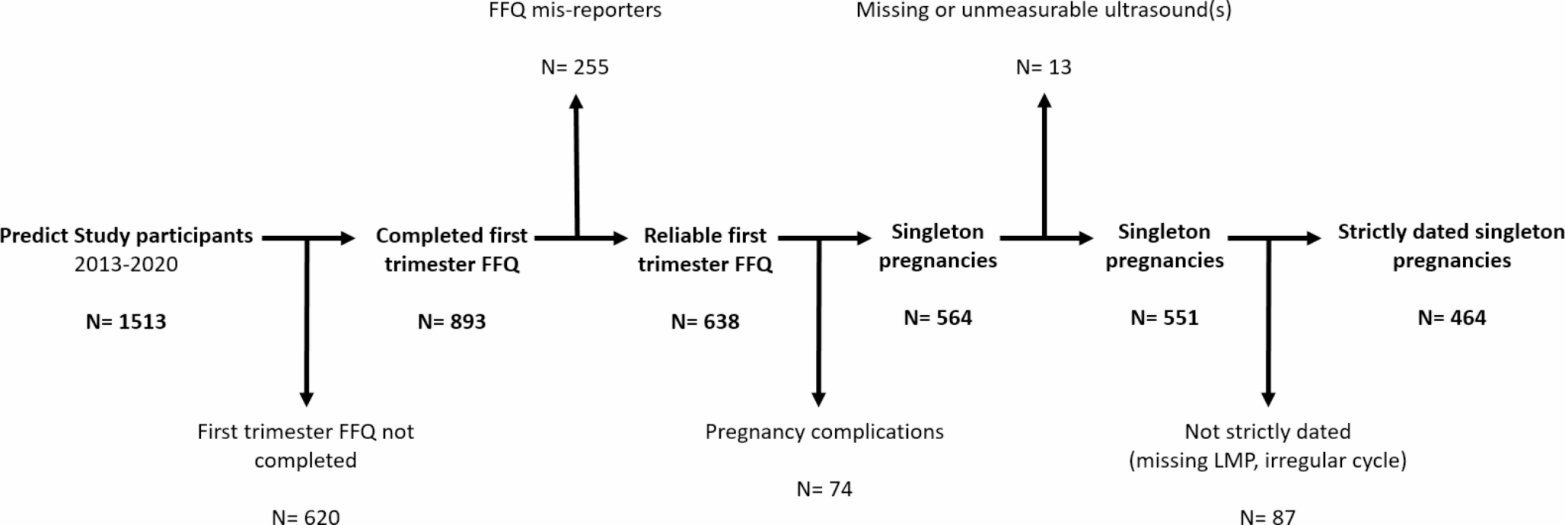
Flowchart of included and excluded populations based on predefined criteria. Pregnancy complications include: miscarriage (*n* = 32), termination of pregnancy (*n* = 8), IUFD (*n* = 2), stillbirth (*n* = 1), postpartum death (*n* = 3), twin pregnancy (*n* = 0), study withdrawal (*n* = 6), congenital abnormalities (*n* = 22). FFQ, food frequency questionnaire; IUFD, intrauterine fetal death; LMP, last menstrual period

Pregnant women in early first trimester (< 8 weeks of gestation) were eligible for enrolment provided they (and their partner) had at least reached the age of 18 years, and could read and speak the Dutch language. Pregnancies achieved by natural conception (including intrauterine insemination, IUI) and assisted reproductive techniques (in vitro fertilization, IVF, intracytoplasmic sperm injection, ICSI) were included. Figure 1 shows the criteria for inclusion and exclusion for the current study. Women were included if embryonic measurements were available, from September 2013 to December 2020 [39, 40]. If a woman participated more than once, only data from the first inclusion was used.

### Dietary intake

At enrollment, between 6 and 13 weeks of gestation, all participants received a standardized food frequency questionnaire (FFQ) developed at the Division of Human Nutrition, Wageningen University, the Netherlands [41]. Each FFQ included questions on frequency of food consumption, portion sizes and cooking methods followed in the previous four weeks. A total of 196 food and beverage items were used to determine energy intake, macronutrient and micronutrient content of the diet. The protocols for this calculation have been previously validated based on high correlation coefficients between nutrient intakes derived from the FFQs and the dietary 24-hour recalls with the corresponding nutritional biomarker in blood [42, 43]. In this article, the variables used from the FFQ to determine daily calorie intake and fat intake from the diet during the periconception period include: total energy (total kcal/day), total fat (total fat g/day), saturated FA (g/day), trans FA (g/day), monounsaturated FA (MUFA g/day), polyunsaturated FA (PUFA g/day), linoleic acid (LA g/day), alpha-linoleic acid (ALA g/day), docosahexaenoic acid (DHA g/day) and eicosapentaenoic acid (EPA g/day). Recommended daily intakes of total fat and saturated FA [44], PUFAs [45] and EPA and/or DHA [36] for women in their first trimester of pregnancy were taken from government websites or from the literature. Under-reporters of energy intake were identified and excluded according to the Goldberg cut-off criteria [46]. A comprehensive description of this method can be found in the recent publication of Smit et al. 2022 [41].

### Participant measurements

Gestational age (GA) was calculated from the first day of the last menstrual period of a regular menstrual cycle. For pregnancies achieved by IVF and ICSI, GA was calculated from 14 days before the conception date for fresh embryo transfers and 14 days before the fictitious conception date for cryopreserved embryo transfers, adjusted for the age of the embryo at transfer. For pregnancies achieved via IUI, GA was calculated from 14 days before the insemination date. GA was considered unreliable in cases with an unknown last menstrual period or an irregular menstrual cycle (< 21 or > 35 days) (Fig. 1) [47]. Body mass index (BMI) was calculated (kg/m^2^) at the intake appointment by medical researchers, and participants were classified as: underweight (BMI < 18.5), normal weight (BMI 18.5–24.9), overweight (BMI 25.0-29.9) and obese (BMI ≥ 30). Information on women and their partners was obtained via self-reported questionnaires given at the time of enrolment, covering details such as age, anthropometrics, family history, medical history, education, ethnicity, lifestyle behavior and habits (such as smoking habits, alcohol use, folic acid supplement use, multivitamin use, fish oil supplement use and physical activity) during the periconception period (from 14 weeks before to 10 weeks after conception) [47]. Information on pregnancy sickness was limited and therefore not included in this article. Neonatal data and information on previous pregnancies was obtained from medical records.

### Embryonic measurements

**Fig. 2:**
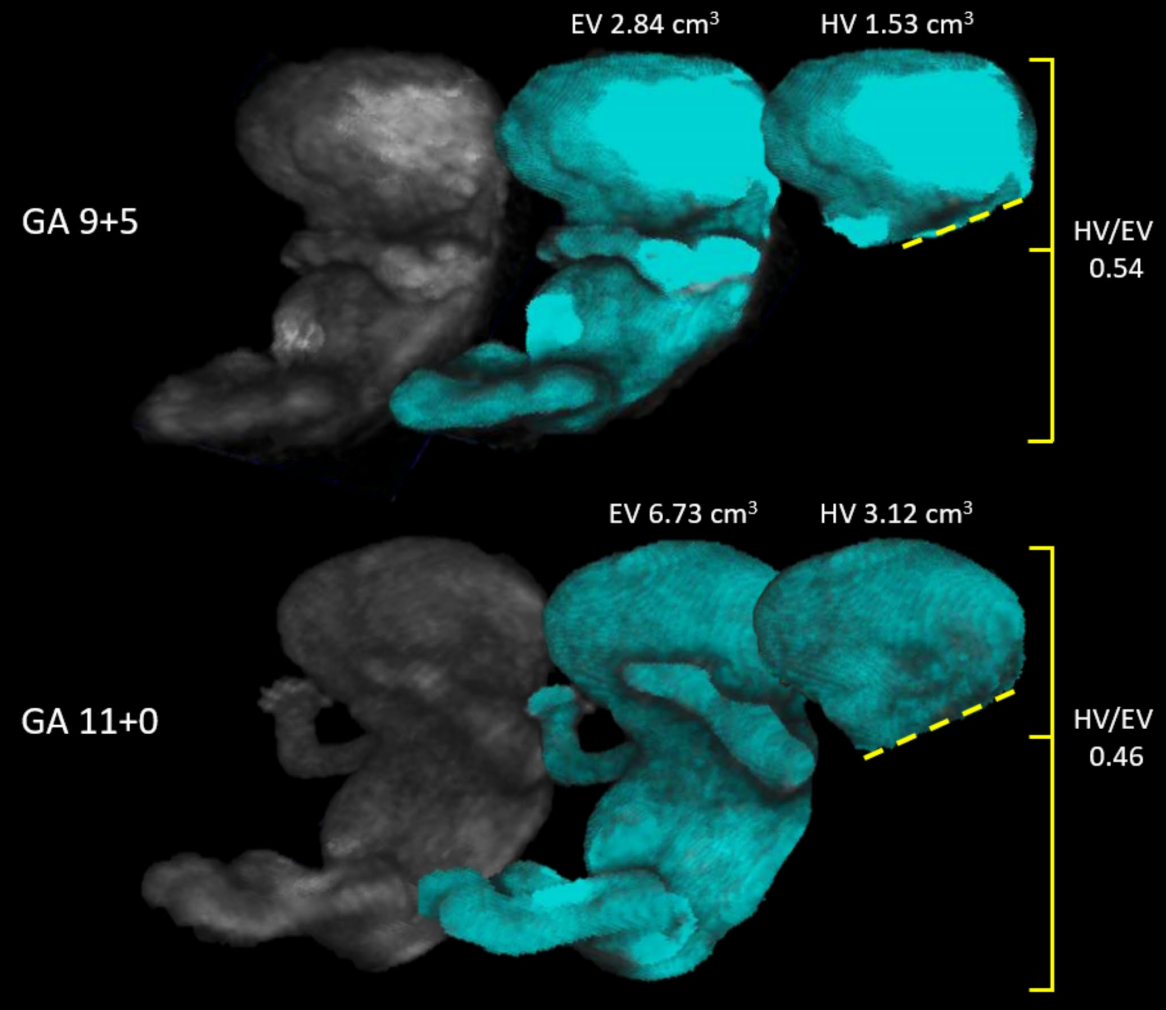
Embryonic measurements performed using Virtual Reality. The process of obtaining volumetric measurements is shown on a 9 and 11-week embryo, from left to right. After manual segmentation (shown in grey), semi-automated volumetric measurements of the whole embryo (EV) and the head (HV) are performed using the V-scope software (shown in blue). The HV/EV ratio is shown on the right. The 2D images shown here are a snapshot of the 3D holograms seen using Virtual Reality. EV, embryonic volume; GA, gestational age; HV, head volume

Longitudinal three-dimensional (3D) ultrasound examinations were performed from enrolment to the end of the first trimester of pregnancy with a maximum of 6 scans per pregnancy. At the beginning of the study, 6 scans were performed, but the pilot study revealed that the number could be reduced to 3 for accurate modelling of embryonic curves [38]. 3D-ultrasound examinations were performed using a 6–12 MHz transvaginal probe of the Voluson E8 or E10 system (General Electric Medical Systems), and the images were analyzed by virtual reality (VR) systems as described in previous studies [47–49]. Previous studies have shown excellent intra and inter-observer reliability of the volumetric measurements [50–53]. Embryonic volume (EV) was performed from 6^+ 0^ to 13^+ 6^ weeks of gestation (Fig. 2). Head volume (HV) was performed at 9 (9^+ 0^-9^+ 6^) and 11 (11^+ 0^-11^+ 6^) weeks of gestation because the required reference points to perform such measurements are not visible earlier or only visible in a small proportion of the included population. To calculate the size of the embryonic head relative to the body, a ratio was obtained (HV/EV ratio) (Fig. 2).

### Statistical analyses

Non-parametric measurements of embryonic volumes were cubic root-transformed to obtain parametric data. Correlation matrices were obtained among the maternal baseline characteristics and dietary intakes. Linear mixed models were used to analyze associations between dietary intakes/patterns and longitudinal measurements of embryonic growth, adjusting for confounders, and assuming the effect of diet was constant over gestation. A spline function was used for GA. For all analyses, confounders were chosen based on the literature [6, 17, 35, 37, 41, 54–62] and the correlation matrix (Results paragraph 2). To increase precision of the estimate, we adjusted for the potential measurer effect and time effect of the ultrasound images. As the transformation scales of the outcome variables (cubic root transformed EV and HV) may be unintuitive for interpretation, we created an effect plot at the original scale, showing the expected embryonic measurements based on the gestational age for different levels for the exposure. Because mathematically, the expectation on the original scale does not equal the back-transformed value of the expectation according to the estimated model, we had to compute the marginal (i.e. population average) effect of diet on embryonic growth in another way. We therefore derived the distribution of the effect of diet on outcome by computing it for a sample of the population sampling subject specific effects according to our model and covariate values (using the distribution in our sample). To take the model uncertainty into account we also sample the fixed effects used for prediction from the multivariate normal distribution of the estimates. These individual subject-specific effects can now be averaged to obtain the marginal (population-average) effect of diet. Allometric relationships between HV and EV were performed, the coefficient of determination (R^2^) was calculated, and differences in the regression lines per subgroup were analyzed with the Wald test. Principal component analysis (PCA) with Varimax rotation was applied to identify dietary patterns of FA with the following intakes: saturated FA, trans FA, MUFA, LA, ALA, EPA, DHA and other PUFAs (PUFA-(LA + ALA + EPA + DHA)). Dietary patterns were identified for Eigen value > 1 and labelled based on the highest scores of factor loadings. Statistical significance was defined as p-value < 0.05. All analyses were executed in IBM SPSS statistics for Windows (version 25) and R (version 4.0.3).

## Results

### Baseline characteristics of the study population

A total of 464 pregnancies were included in the study, as described in Fig. 1. The majority of women were of Western origin and highly educated (Table 1). Compliance to folic acid supplement use was almost total, however multivitamins were consumed in 71% of the population, and fish oil supplements were consumed by 25% of the population (Table 1). Table S1 represents the dietary intake of FA per day of the total study population during early pregnancy, stratified for categories of BMI. Due to limited sample sizes per BMI subgroup, stratification in following analyses was not performed.

**Table 1:** Baseline characteristics of the total study population (*n* = 464)

**Demographics**	
Age at conception, *years*	32.67 ± 3.34
BMI, *kg/m*^*2*^	
Underweight Normal weight Overweight Obese	11 (2.4) 261 (56.3) 140 (30.2) 52 (11.2)
Ethnicity, *n (%)*	
Western Non-western	397 (88.8) 50 (11.2)
Education level, *n (%)*	
Low Intermediate High	32 (7.2) 136 (30.4) 279 (62.4)
**Lifestyle (periconception period)**	
Alcohol use, *n (%)*	133 (29.8)
Smoking, *n (%)*	55 (12.3)
Folic acid supplement use, *n (%)*	445 (99.6)
Multivitamin use, *n (%)*	318 (71.0)
Fish oil use, *n (%)*	110 (25.0)
Physical activity, *n (%)*	215 (48.0)
**Pregnancy**	
Nulliparous, *n (%)*	175 (39.1)
Conception mode, *n (%)*	
Natural IVF/ICSI Frozen ET Fresh ET	229 (49.4) 235 (50.6) 85 (50.5) 149 (32.2)
Gestational age at birth, *days*	271 ± 13.84
Birth weight, *grams*	3268.27 ± 551.61

Data are represented as mean ± SD or n (%). n represents the number of individuals that fall into that variable category. Percentages are represented excluding the missing values (< 20%). BMI was classified as: underweight < 18.5, normal weight 18.5–24.9, overweight 25.0-29.9 and obese ≥ 30. BMI, body mass index; ET, embryo transfer; ICSI, intracytoplasmic sperm injection; IVF, in vitro fertilization

### Maternal characteristics and dietary intakes

In this population, women that were older, exercised and were in their first pregnancy had a higher dietary intake of EPA (age *r* = 0.114, *p* = 0.014; exercise *r* = 0.189, *p* < 0.001; parity *r* = 0.117, *p* = 0.013) and DHA (age *r* = 0.115, *p* = 0.013; exercise *r* = 0.178, *p* < 0.001; parity *r* = 0.111, *p* = 0.019). Maternal smoking did not correlate with dietary FA intake, nor BMI, but alcohol consumers had a higher dietary intake of EPA (*r* = 0.127, *p* = 0.007) and DHA (*r* = 0.101, *p* = 0.032). The use of folic acid, multivitamin or fish oil supplement was not correlated with dietary FA intake.

### Fatty acid intake and embryonic growth

To study associations between maternal dietary intake of FA and repeated measurements of embryonic growth in the first trimester, linear mixed models were used adjusting for relevant confounders. The dietary intake of total fat, saturated FA, trans FA, MUFA, PUFA, LA and ALA measured as 10 g per day was not associated with changes in EV, HV nor in the HV/EV ratio in the first trimester (Table 2). This trend is shown in the effect plots of Figure S1, where EV and HV are represented in their original scale. However, after adjusting for confounders, a higher dietary intake of EPA, DHA and other PUFAs was associated with a smaller HV/EV ratio (Table 2).

**Table 2:** Linear mixed models between maternal FA intake and longitudinal first trimester embryonic measurements in the total population (*n* = 464)

	√^3^EV	√^3^HV	HV/EV
**Total fat**	*N* = 416	*N* = 361	*N* = 344
Model 1	-0.001 (-0.007,0.004), *p* = 0.636	0.002 (-0.001,0.006), *p* = 0.261	-0.001 (-0.003,1.970^e − 04^), *p* = 0.081
Model 2	-4.839^e − 03^ (-1.595^e − 02^,6.275^e − 03^), *p* = 0.392	1.559^e − 03^ (-7.283^e − 03^,1.040^e − 02^), *p* = 0.728	-1.595^− 03^ (-4.911^e − 03^,1.719^e − 03^), *p* = 0.344
**Saturated FA**	*N* = 416	*N* = 361	*N* = 344
Model 1	-0.002 (-0.015,0.011), *p* = 0.773	0.007 (-0.003,0.016), *p* = 0.166	-0.002 (-0.006,0.002), *p* = 0.334
Model 2	-6.940^e − 03^ (-3.004^e − 02^,1.616^e − 02^, *p* = 0.554	4.385 ^− 03^ (-1.379^e − 02^,0.023), *p* = 0.635	8.568^e − 04^ (-0.006,7.828^e − 03^), *p* = 0.809
**Trans FA**	*N* = 349	*N* = 292	*N* = 283
Model 1	0.054 (-0.187,0.294), *p* = 0.662	**0.216 (0.038**,**0.395)**, ***p*** = **0.017**	-0.003 (-0.081,0.075), *p* = 0.935
Model 2	8.723^e − 02^ (-2.977^e − 01^,4.722^e − 01^), *p* = 0.655	2.911^e − 01^ (-1.938^e − 03^,5.843^e − 01^), *p* = 0.051	8.691^e − 02^ (-0.028,2.018^e − 01^), *p* = 0.137
**MUFA**	*N* = 416	*N* = 361	*N* = 344
Model 1	-0.006 (-0.021,0.009), *p* = 0.472	0.003 (-0.009,0.015), *p* = 0.616	**-0.006 (-0.011**,**-6.769**^**e − 04**^**)**, ***p*** = **0.026**
Model 2	-1.370^e − 02^ (-4.098^e − 02^,1.357^e − 02^), *p* = 0.324	-5.523^e − 03^ (-2.773^e − 02^,1.669^e − 02^), *p* = 0.624	-7.655^e − 03^ (-1.583^e − 02^,5.238^e − 04^), *p* = 0.066
**PUFA**	*N* = 416	*N* = 361	*N* = 344
Model 1	-0.003 (-0.026,0.019), *p* = 0.769	0.008 (-0.008,0.025), *p* = 0.322	-0.006 (-0.013,0.001), *p* = 0.094
Model 2	-8.331^e − 03^ (-3.985^e − 02^,2.319^e − 02^), *p* = 0.603	7.907^e − 03^ (-1.698^e − 02^,3.279^e − 02^), *p* = 0.532	-3.617^e − 03^ (-0.013,5.433^e − 03^), *p* = 0.431
**LA**	*N* = 416	*N* = 361	*N* = 344
Model 1	-0.004 (-0.031,0.022), *p* = 0.736	0.011 (-0.009,0.030), *p* = 0.301	-0.005 (-0.013,0.003), *p* = 0.197
Model 2	-1.119^e − 02^ (-4.724^e − 02^,2.484^e − 02^), *p* = 0.541	8.677^e − 03^ (-1.980^e − 02^,3.715^e − 02^), *p* = 0.549	-1.420^e − 03^ (-1.185^e − 02^,9.018^e − 03^), *p* = 0.789
**ALA**	*N* = 416	*N* = 361	*N* = 344
Model 1	0.026 (-0.189,0.242), *p* = 0.808	0.108 (-0.057,0.273), *p* = 0.202	-0.042 (-0.105,0.022), *p* = 0.204
Model 2	-9.600^e − 03^ (-2.980^e − 01^,0.278), *p* = 0.947	1.224^e − 01^ (-1.058^e − 01^,3.507^e − 01^), *p* = 0.292	-7.082^e − 03^ (-8.809^e − 02^,7.392^e − 02^), *p* = 0.863
**EPA**	*N* = 416	*N* = 361	*N* = 344
Model 1	-0.852 (-2.511,0.807), *p* = 0.313	-0.580 (-1.982,0.821), *p* = 0.415	**-0.621 (-1.190**,**-0.051)**, ***p*** = **0.032**
Model 2	-6.842^e − 01^ (-2.458,1.090), *p* = 0.448	-5.431^e − 01^ (-2.089,1.002), *p* = 0.489	**-6.810**^**e − 01**^**(-1.273**,**-8.905**^**e − 02**^**)**, ***p*** = **0.024**
**DHA**	*N* = 416	*N* = 361	*N* = 344
Model 1	-0.574 (-1.669,0.521), *p* = 0.303	-0.396 (-1.340,0.546), *p* = 0.409	**-0.431 (-0.819**,**-0.044)**, ***p*** = **0.028**
Model 2	-4.150^e − 01^ (-1.588,7.586^e − 01^), *p* = 0.487	-3.416^e − 01^ (-1.388,7.054^e − 01^), *p* = 0.521	**-4.796**^**e − 01**^**(-8.804**^**e − 01**^,**-7.871**^**e − 02**^**)**, ***p*** = **0.018**
**Other PUFAs**	*N* = 416	*N* = 361	*N* = 344
Model 1	0.005 (-0.209,0.220), *p* = 0.962	-0.004 (-0.167,0.159), *p* = 0.963	**-0.106 (-0.170**,**-0.043)**, ***p*** = **0.001**
Model 2	4.811^e − 02^ (-0.187,2.836^e − 01^), *p* = 0.688	8.425^e − 03^ (-1.715^e − 01^,1.884^e − 01^), *p* = 0.926	**-1.155**^**e − 01**^**(-1.825**^**e − 01**^,**-4.862**^**e − 02**^**)**, ***p*** < **0.001**

Represented as Beta (95% CI), p-value. Each dietary intake is expressed in 10 g/day. Other PUFAs = PUFA-(LA + ALA + EPA + DHA). Model 1 adjusted for GA. Model 2 adjusted for GA, total kcal/day, conception mode, BMI, smoking, maternal age, multivitamin use, parity, fetal gender, fish oil supplement, measurer. ALA, alpha-linoleic acid; DHA, docosahexaenoic acid; EPA, eicosapentaenoic acid; EV, embryonic volume; FA, fatty acid; HV, head volume; MUFA, monounsaturated fatty acids; LA, linoleic acid; PUFA, polyunsaturated fatty acids. *P* < 0.05 in bold

To verify the effect of fish oil supplement use on embryonic growth, excluding the influence of dietary fat intake from food, a secondary analysis was performed adjusting for relevant confounders (**Table S2**). The use of fish oil supplement was not associated with EV, HV or HV/EV ratio.

### Fatty acid recommendations and embryonic growth

The total population was categorized according to whether they met, were above or below the recommended dietary intakes of FA during early pregnancy according to health guidelines (**Materials and Methods paragraph 2**). 80.8% (*n* = 375) of the population met the recommended dietary intake of total fat (20–40 E%), 0.4% (*n* = 2) was below and 18.8% (*n* = 87) above. 15.3% (*n* = 71) of the population met the recommended dietary intake of saturated FA (< 10 E%), but 84.7% (*n* = 393) was above. 66.8% (*n* = 310) of the population met the recommended dietary intake of PUFA (6–11 E%), 30.0% (*n* = 139) was below and 3.2% (*n* = 15) was above. 19.0% (*n* = 88) of the population met the recommended dietary intake of EPA and/or DHA (250–450 mg/day), 71.6% (*n* = 332) was below and 9.5% (*n* = 44) was above.

Linear mixed models were performed between maternal dietary FA intakes, using the recommended ranges as reference, and first trimester embryonic growth. **Table S3** shows that only consuming high dietary saturated FA was associated with a smaller HV/EV ratio, and low dietary EPA and/or DHA was associated with a larger HV/EV ratio. **Figure S2** was created to determine the allometric relationship between HV and EV growth per FA dietary intake based on recommended health guidelines. No difference was observed in the Beta coefficient of the slopes among groups, meaning the rate of growth of HV per EV was not associated with recommended dietary intakes of FA.

### Fatty acid patterns and embryonic growth

PCA analysis among dietary FA intakes revealed three uncorrelated FA dietary patterns explaining 87% of the variance of the overall dietary FA intake among the total population (Table 3). The first component was labelled ‘Non-PUFA-rich diet’ explaining the majority of variance (54.9%), followed by the second component labelled ‘DHA and EPA-rich diet’ (21.9% of variance) and the third component ‘PUFA-rich diet’ (10.5% of variance) (Table 3). Outputs from linear mixed models between FA dietary patterns and repeated measurements of embryonic growth in the first trimester are reported in Table 4. A dietary pattern deplete in dietary PUFAs was associated with a larger HV/EV ratio, whereas dietary patterns rich in dietary PUFAs were associated with a smaller HV/EV ratio (Table 4).

**Table 3:** Relation between FA intakes and the identified FA dietary patterns expressed by factor loadings using PCA analysis

		Component 1 ‘Non-PUFA-rich diet’	Component 2 ‘DHA and EPA-rich diet’	Component 3 ‘PUFA-rich diet’
Variance	Individual %	54.87	21.89	10.50
	Cumulative %	54.88	76.77	87.28
FA contribution to dietary patterns	Saturated FA	0.936*	0.122	0.192
	Trans FA	0.928*	0.089	0.084
	MUFA	0.804*	0.196	0.441
	LA	0.599	0.058	0.673*
	ALA	0.604	0.059	0.669*
	EPA	0.123	0.985*	0.083
	DHA	0.114	0.986*	0.083
	Other PUFAs	0.077	0.094	0.817*

The factor loadings describe the FA contribution to each dietary pattern. The factor loadings with the highest absolute value were chosen to describe the pattern (*). Each dietary intake is expressed in g/day. Other PUFAs = PUFA-(LA + ALA + EPA + DHA). ALA, alpha-linoleic acid; DHA, docosahexaenoic acid; EPA, eicosapentaenoic acid; FA, fatty acid; MUFA, monounsaturated fatty acids; LA, linoleic acid; PCA, principal component analysis; PUFA, polyunsaturated fatty acids

**Table 4:** Linear mixed models between FA dietary patterns and longitudinal first trimester embryonic measurements in the total population (*n* = 464)

	√^3^EV	√^3^HV	HV/EV ratio
Non-PUFA-rich diet	*N* = 349	*N* = 292	*N* = 283
	-7.282^e − 04^ (-2.327^e − 02^,2.473^e − 02^), *p* = 0.952	1.752^e − 02^ (-0.001,3.601^e − 02^), *p* = 0.063	**1.031**^**e − 02**^**(2.966**^**e − 03**^,**1.764**^**e − 02**^**)**, ***p*** = **0.006**
DHA and EPA-rich diet	*N* = 349	*N* = 292	*N* = 286
	-3.453^e − 03^ (-0.0.018,1.065^e − 02^), *p* = 0.630	-5.800^e − 03^ (-1.812^e − 02^,6.521^e − 03^), *p* = 0.354	**-4.917**^**e − 03**^**(-9.938**^**e − 03**^,**1.027**^**e − 04**^**)**, ***p*** = **0.054**
PUFA-rich diet	*N* = 349	*N* = 292	*N* = 283
	-7.404^e − 04^ (-0.015,1.395^e − 02^), *p* = 0.921	-7.160^e − 03^ (-1.815^e − 02^,3.830^e − 03^), *p* = 0.203	**-6.783**^**e − 03**^**(-1.098**^**e − 02**^,**-2.582**^**e − 03**^**)**, ***p*** = **0.002**

Represented as Beta (95% CI), p-value. Model 1 adjusted for gestational age. Only shown is Model 2 adjusted for gestational age, total kcal/day, conception mode, BMI, smoking, maternal age, multivitamin use, parity, fetal gender, fish oil supplement, measurer. EV, embryonic volume; PUFA, polyunsaturated fatty acids; HV, head volume. *P* < 0.05 in bold

## Discussion

The current study reports associations between maternal dietary PUFA intake and first trimester embryonic body and head growth. More than 70% of the population was below the recommended dietary intake of EPA and/or DHA. Although maternal total FA, saturated FA, trans FA, MUFA and total PUFA dietary intakes were not associated with changes in embryonic growth, we observed that a higher dietary intake of EPA, DHA (and a combination of omega 3 and 6 PUFAs more generally) was associated with a smaller embryonic HV/EV ratio. Also, omega 3 FA from fish oil supplement was not associated with a smaller HV/EV ratio. Similarly, we found that compliance to a dietary pattern rich in PUFAs and/or omega 3 was associated with a smaller embryonic HV/EV ratio.

The HV/EV ratio is a relative measurement that describes the proportion between body structures, giving more insight into growth patterns, instead of growth in absolute terms. The relationship between the head and the body is allometric, indicating it changes over time. From the fetal period to adulthood, the body grows at a faster speed relative to the head [63]. Several published normograms for prenatal fetal growth show that the head-to-body ratio decreases in linear fashion during gestation [64]. An asymmetric and higher head-to-body ratio is associated with adverse pregnancy outcomes, such as preterm birth, low birth weight and fetal distress; meaning that the fetus fails to reach its optimal growth potential [64, 65]. Therefore, a positive fetal growth potential and optimal growth symmetry are represented by the negative association between dietary DHA, EPA and other PUFAs and HV/EV ratio in the current study. This is supported by the findings of Vafai et al. (2022), according to which DHA and EPA were associated with a smaller medial head circumference-to-abdominal circumference ratio in the second and third trimesters of pregnancy [30].

In the current study, dietary omega 3 and 6 FA, but not omega 3 FA from fish oil supplements specifically, was associated with a smaller HV/EV ratio. This difference could be explained by the degree of uptake of omega 3 and 6 by tissues, which has a more pronounced influence on pregnancy and embryonic tissues. Compared to fish oil capsules only, which are composed of DHA and EPA solely, omega 3-rich foods, such as fatty fish, are rich in a range of nutrients and a complex FA profile. The co-ingestion of DHA and EPA with other fats has been shown to significantly facilitate their cellular absorption [66, 67]. It can also be argued that the nutritional composition of PUFA-rich foods is beneficial and contributes to fetal growth, which is lacking in fish oil capsules. Foods high in PUFAs, such as nuts, also contain important amino acids and fiber, known to confer multiple health benefits overall and during pregnancy. We interpret that consumption of omega 3 and 6 from dietary sources is more relevant for pregnancy than consumption of fish oil capsules.

Being born with a low or high birth weight can influence postnatal body fat distribution and increase the risk of developing obesity and chronic diseases in later life [68–70]. High consumption of fats during pregnancy is associated with increased fetal adiposity and low birth weight [71, 72]. In this cohort, where the majority of the population was within the recommended intake of total fat, we did not find any association between maternal total FA intake and first trimester embryonic growth measurements. After breaking down the FA into subgroups, this association did not change. According to the literature, consumption of saturated and trans FA is associated with fetal growth restriction and large for gestational age offspring but, consistent with the study from Cohen et al. (2011), an effect on embryonic size, representative of first trimester of pregnancy, may not be observable [20, 73]. For trans FA specifically, our results may differ from those published previously as the maximum level of industrially produced trans FA in food products has, in recent years, been strictly regulated by EU regulations [74].

Based on the knowledge that PUFAs are critical for neurodevelopment and brain connectivity, it might be considered unusual to find no associations between PUFA dietary intake and embryo head volume. Head volume is indicative of brain development and maturation; and smaller head size and slower head growth are associated with neurodevelopmental disorders [75, 76]. These results may be due to the embryo stages assessed in the current study (first trimester), whereas the second and third trimesters are the key time periods for fetal adipose tissue metabolic and neural programming. However, we cannot exclude the possibility that FA intake is associated with changes in growth, metabolism and brain development at both cellular and molecular levels [77–79].

Lastly, in our population, compliance with fish oil supplement use amounted to only 25% of the population, which was not correlated to the amount of dietary fat consumed, whether total or specifically PUFAs, despite > 70% of the population being below the recommended intake of EPA and/or DHA. This mirrors reports for most western populations (including the United States) and reflects the current lack of awareness on the benefits of consuming fish oil supplements during pregnancy [36, 80, 81]. The Health Council of the Netherlands recommends pregnant women to eat fish twice a week, and take fish oil supplements if women cannot or do not want to eat this amount of fish [82]. Unlike folic acid, there is insufficient evidence supporting the benefits of taking fish oil supplements during pregnancy. Additionally, the higher cost of the product, whether fish oil supplement alone or in combination with a multivitamin, may discourage women from taking fish oil supplements.

### Clinical implications

Maternal dietary intake of omega 3 and 6 FA influences embryo growth and fetal body proportions, which may reduce the risk of pregnancy complications [29, 83]. Most importantly, we found that the embryo is sensitive to such nutrient intakes as early as the first weeks of pregnancy, which supports the importance of maternal nutrition around the periconceptional period. Furthermore, we stress that dietary sources of omega 3 and 6 FA (not specifically derived from fish oil supplements) are associated with embryonic growth. As the majority of women do not meet the recommended dietary intakes of omega 3 and 6, we suggest that pregnancy care should implement advice on the appropriate consumption of omega 3 and 6-rich whole foods to support fetal growth.

### Research implications

In this study, we were able to show that maternal dietary PUFAs influence embryonic growth, but unable to demonstrate that dietary PUFAs are crucial for prenatal brain development. A suggestion for future studies would be the use of more detailed scans of brain structures, which would add more in-depth information compared to HV. We were also unable to interpret if altered HV/EV ratios would also have implications on postnatal neurodevelopment. To our knowledge, most literature focuses on absolute measurements of embryonic head (head volume and circumference) and brain size (e.g. intracranial structure and fissure sizes), and there is limited understanding on brain development in proportion to body size and growth patterns. Knowing that smaller brain structures, especially during early pregnancy, are associated with poor cognitive, language and motor tests in early infancy [40, 84], we hypothesize that HV/EV measurements are relevant to understand postnatal neurodevelopment. We conclude that research is needed to study embryonic and fetal brain structures in proportion to body size, and in association with postnatal neurodevelopment. Additionally, future studies should consider the efficacy of fish oil supplement consumption in combination with fatty-rich diets on embryonic growth.

### Strengths and limitations

This is the first study to analyze the effects of dietary FA intake and fish oil supplement use on repeated measurements of embryonic growth. To date, the majority of studies include later stages of gestation, or early postnatal growth measurements. All included repeated measurements have previously shown to have excellent inter- and intra-observer reproducibility [37]. Lastly, the VR approach of performing 3D embryonic measurements substantially improves the precision of measurements as opposed to 2D measurements. The use of estimated dietary intakes by means of FFQs, instead of collecting biological concentrations, was a limitation of this study, as well as the unknown dose of fish oil supplements. Measurements of embryo HV and EV, despite being a proxy for embryo brain growth and overall metabolism, can’t reveal the cellular or molecular changes that may occur.

## Conclusion

Dietary intake of PUFAs could be associated with early embryonic development. Due to the high prevalence of omega 3-deficient pregnant women, dietary intake of PUFAs during the periconceptional period should be encouraged by health care professionals to reduce risks of pregnancy complications.

## Electronic supplementary material

Below is the link to the electronic supplementary material.

Supplementary Material 1
