# Supplementary material for: Maternal fatty acid intake and human embryonic growth: the Rotterdam Periconception Cohort
Source: Eur J Epidemiol. 2024 Dec 11;39(12):1379–89. doi: 10.1007/s10654-024-01184-8 (PMC11680660; doi:10.1007/s10654-024-01184-8)
Supplement: Supplementary file 1 — Supplementary Material 1 [file 10654_2024_1184_MOESM1_ESM.docx]

**Journal name:** European Journal of Epidemiology

**Title:** Maternal fatty acid intake and human embryonic growth: The Rotterdam Periconception Cohort

**Author list:** Eleonora RUBINI^1^, Lenie VAN ROSSEM^1,4^, Sam Schoenmakers^1^, Sten P. Willemsen^1,2^, Kevin D. Sinclair^3^, Régine P.M. Steegers-Theunissen^1*^, Melek Rousian^1^

**Affiliations:**

^1^Department of Obstetrics and Gynecology, Erasmus MC, University Medical Centre, Rotterdam, The Netherlands.

^2^Department of Biostatistics, Erasmus MC, University Medical Center, Rotterdam, The Netherlands.

^3^School of Biosciences, University of Nottingham, Sutton Bonington, Leicestershire, UK.

^4^Division of Human Nutrition and Health, Wageningen University, Wageningen, The Netherlands

*Corresponding author. Department of Obstetrics and Gynecology, Erasmus MC, University Medical Center, PO Box 2040, Room Ee2271a, 3000 CA Rotterdam, The Netherlands. Tel: +31 10 7038254 | +31 6 12 47 26 43. Email: [r.steegers@erasmusmc.nl](mailto:r.steegers@erasmusmc.nl)

**Table S1.** Dietary intake of macronutrients of the total study population and per BMI categories.

|  | Total study population  (n= 464) | BMI | | | |
| --- | --- | --- | --- | --- | --- |
|  |  | **Underweight**  **(n=11)** | **Normal weight**  **(n=261)** | **Overweight**  **(n=140)** | **Obese**  **(n=52)** |
| Energy intake, *kcal/day* | 1922.13 ± 560.864 | 1881.40 ± 718.82 | 1882.65 ± 476.74 | 1952.10 ± 660.37 | 2048.16 ± 616.60 |
| Total fat, *E%/day*  Saturated fatty acids  Trans fatty acids  MUFA  PUFA  LA  ALA  EPA  DHA | 35.23 ± 5.41  12.62 ± 2.67  0.59 ± 1.77  12.56 ± 2.32  7.02 ± 1.85  5.75 ± 1.63  0.76 ± 0.20  0.03 (0.01-0.05)  0.04 (0.01-0.08) | 33.67 ± 4.65  11.11 ± 1.47  0.51 ± 0.16  11.92 ± 2.43  7.74 ± 1.81  6.44 ± 1.45  0.82 ± 0.20  0.04 (0.01-0.09)  0.04 (0.01-0.14) | 35.19 ± 5.44  12.62 ± 2.63  0.58 ± 0.17  12.58 ± 2.29  6.97 ± 1.90  5.69 ± 1.68  0.75 ± 0.20  0.03 (0.01-0.06)  0.04 (0.01-0.08) | 35.89 ± 4.89  12.88 ± 2.52  0.63 ± 0.19  12.87 ± 2.24  7.06 ± 1.77  5.82 ± 1.59  0.76 ± 0.18  0.03 (0.01-0.06)  0.04 (0.02-0.08) | 33.91 ± 6.46  12.20 ± 3.33  0.56 ± 0.17  11.75 ± 2.51  6.96 ± 1.81  5.69 ± 1.52  0.76 ± 1.20  0.02 (0.01-0.05)  0.02 (0.01-0.06) |

Data are represented as mean ± SD or n (%) or median (interquartile range). Percentages are represented excluding the missing values (<20%). ALA, alpha linoleic acid; BMI, body mass index; DHA, docosahexaenoic acid; E%, percentage from energy intake; EPA, eicosapentaenoic acid; LA, linoleic acid; MUFA, monounsaturated fatty acids; PUFA, polyunsaturated fatty acids.


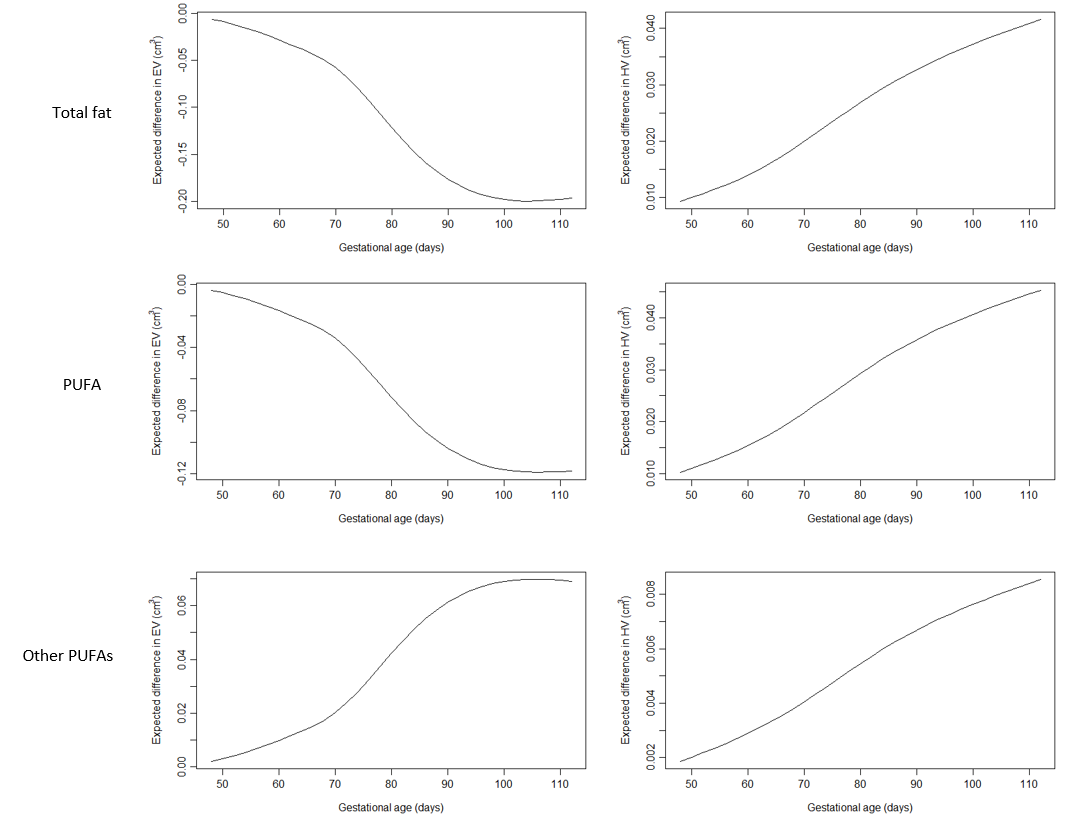

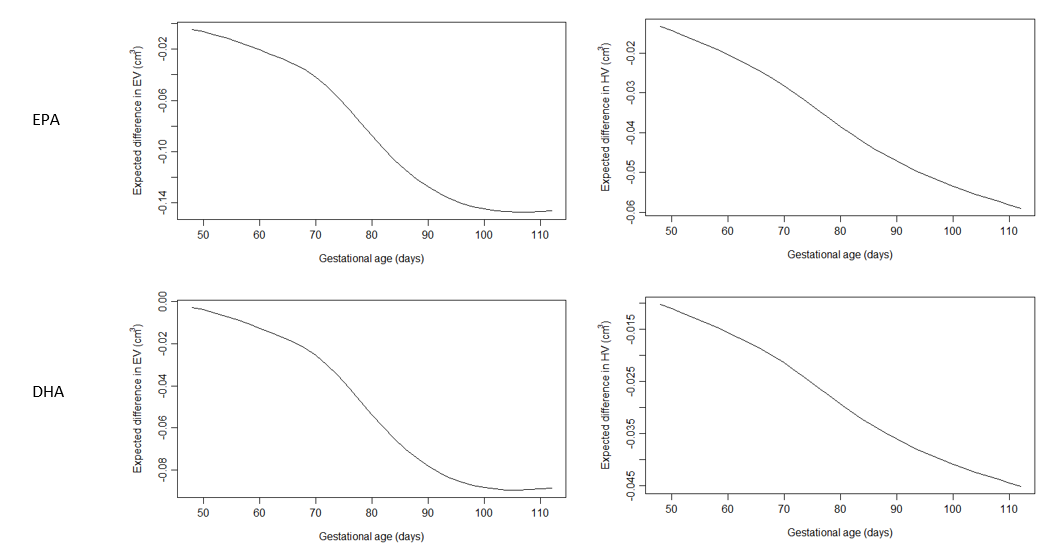


**Figure S1.** Effect plot representing the average (expected) difference in embryonic volumes per gestational day when the intake of fatty acids is in the highest quartile compared to the lowest. DHA, docosahexaenoic acid; EPA, eicosapentaenoic acid; EV, embryonic volume; HV, head volume; PUFA, polyunsaturated fatty acids.

**Table S2.** Linear mixed models between maternal fish oil supplement use and longitudinal first trimester embryonic measurements in the total population (n= 464).

|  | √^3^EV | √^3^HV | HV/EV |
| --- | --- | --- | --- |
| Fish oil supplement use | N= 416 | N= 361 | N= 344 |
| Model 1 | **0.0298 (0.000,0.059), p=0.0.048** | 0.005 (-0.018,0.028), p=0.688 | -0.002 (-0.012,0.006), p=0.575 |
| Model 2 | 2.305^e-02^ (-7.599^e-03^,5.371^e-02^), p=0.139 | -3.950^e-03^ (-2.812^e-02^,0.020), p=0.747 | -4.358^e-04^ (-9.607^e-03^,8.756^e-03^), p=0.927 |
| Multivitamin use | N= 425 | N= 368 | N= 350 |
| Model 1 | **0.028 (0.000,0.056), p=0.048** | 0.019 (-0.004,0.041), p=0.109 | -0.000 (-0.009,0.008), p=0.918 |
| Model 2 | 0.027 (-1.350^e-03^,5.609^e-02^), p=0.062 | 1.967^e-02^ (-3.744^e-03^,4.310^e-02^), p=0.099 | -1.178^e-03^ (-9.847^e-03^,0.007), p=0.789 |

Represented as Beta (95% CI), p-value. Model 1 adjusted for GA. Model 2 adjusted for GA, total kcal/day, conception mode, total fat intake, BMI, smoking, maternal age, parity, fetal gender, measurer. EV, embryonic volume; HV, head volume. P<0.05 in bold.

**Table S3.** Linear mixed models between maternal FA intakes according to health recommendations and first trimester embryonic growth.

|  | **√^3^EV** | | **√^3^HV** | | **HV:EV** | |
| --- | --- | --- | --- | --- | --- | --- |
|  | В (95% CI) | P-value | В (95% CI) | P-value | В (95% CI) | P-value |
| **Total fat** | | | | | | |
| 20-40 E% | reference | | reference | | reference | |
| >40 E% | -2.097^e-02^ (-5.382^e-02^,1.187^e-02^) | 0.209 | 3.074^e-04^ (-2.636^e-02^,0.027) | 0.982 | 6.435^e-03^ (-3.665^e-03^,1.653^e-02^) | 0.212 |
| **Saturated FA** | | | | | | |
| <10 E% | reference | | reference | | reference | |
| >10 E% | 9.286^e-03^ (-2.649^e-02^,4.506^e-02^) | 0.609 | -9.602^e-03^ (-3.785^e-02^,0.018) | 0.504 | **-1.280^e-02^ (-2.358^e-02^,-2.023^e-03^)** | **0.020** |
| **PUFA** | | | | | | |
| <6 E% | 8.205^e-03^ (-1.977^e-02^,0.036) | 0.564 | -1.674^e-03^ (-2.026^e-02^,0.023) | 0.881 | 2.625^e-03^ (-6.141^e-03^,1.139^e-02^) | 0.556 |
| 6-11 E% | reference | | reference | | reference | |
| > 11 E% | **-6.801^e-02^ (-1.352^e-01^,7.954^e-03^)** | **0.048** | -3.126^e-02^ (-8.966^e-02^,2.714^e-02^) | 0.293 | 1.421^e-04^ (-2.173^e-02^,2.201^e-02^) | 0.989 |
| **EPA+DHA** | | | | | | |
| <250 mg/day | -1.447^e-02^ (-0.048,1.942^e-02^) | 0.401 | -3.265^e-03^ (-3.069^e-02^,0.024) | 0.815 | **1.169^e-02^ (1.409^e-03^,2.197^e-02^)** | **0.026** |
| 250-450 mg/day | reference | | reference | | reference | |
| >450 mg/day | -3.912^e-02^ (-8.124^e-02^,2.991^e-03^) | 0.066 | -8.431^e-03^ (-5.074^e-02^,3.388^e-02^) | 0.692 | -1.626^e-03^ (-1.803^e-02^,1.477^e-02^) | 0.844 |

Represented as Beta (95% CI), p-value. Shown is Model 2 adjusted for GA, total kcal/day, conception mode, BMI, smoking, maternal age, multivitamin use, parity, fetal gender, fish oil supplement, measurer. DHA, docosahexaenoic acid; EPA, eicosapentaenoic acid; FA, fatty acid; PUFA, polyunsaturated fatty acids. P<0.05 in bold.


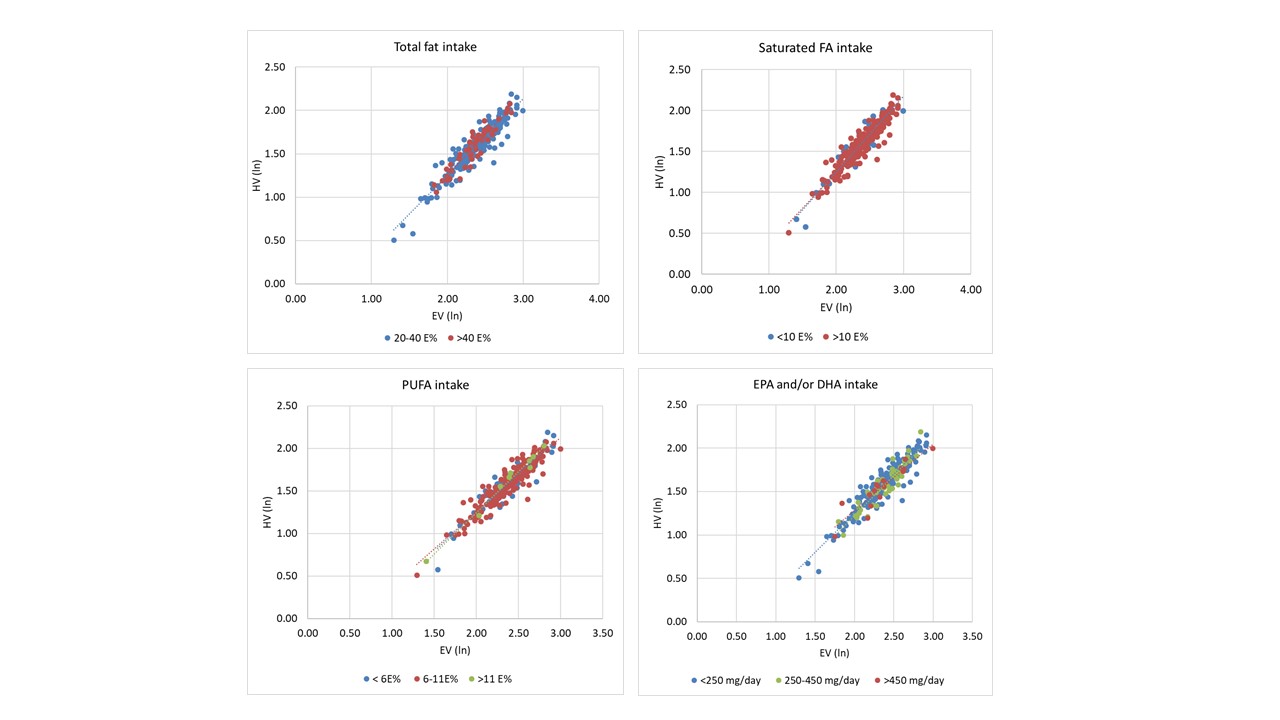


**Figure S2.** Allometric relationships between HV and EV at 11 weeks GA stratified by recommended intakes of FA. HV and EV were transformed to the natural logarithm (ln) to obtain linearity. Total fat intake 20-40 E% R^2^=0.877, >40 E% R^2^=0.885. Saturated FA <10 E% R^2^=0.879, >10 E% R^2^= 0.876. PUFA <6 E% R^2^=0.870, 6-11 E% R^2^= 0.867, >11 E% R^2^= 0.985. EPA and/or DHA < 250mg/day R^2^=0.883, 250-450mg/day R^2^= 0.865, >450mg/day R^2^= 0.852. DHA, docosahexaenoic acid; EPA, eicosapentaenoic acid; EV, embryonic volume; FA, fatty acids; HV, head volume; PUFA, polyunsaturated fatty acids.
